# DockQ v2: improved automatic quality measure for protein multimers, nucleic acids, and small molecules

**DOI:** 10.1093/bioinformatics/btae586

**Published:** 2024-09-30

**Authors:** Claudio Mirabello, Björn Wallner

**Affiliations:** Division of Bioinformatics, Department of Physics, Chemistry and Biology, Linköping University, SE-581 83 Linköping, Sweden; National Bioinformatics Infrastructure Sweden, Science for Life Laboratory, Linköping University, SE-581 83 Linköping, Sweden; Division of Bioinformatics, Department of Physics, Chemistry and Biology, Linköping University, SE-581 83 Linköping, Sweden

## Abstract

**Motivation:**

It is important to assess the quality of modeled biomolecules to benchmark and assess the performance of different prediction methods. DockQ has emerged as the standard tool for assessing the quality of protein interfaces in model structures against given references. However, as predictions of large multimers with multiple chains become more common, DockQ needs to be updated with more functionality for robustness and speed. Moreover, as the field progresses and more methods are released to predict interactions between proteins and other types of molecules, such as nucleic acids and small molecules, it becomes necessary to have a tool that can assess all types of interactions.

**Results:**

Here, we present a complete reimplementation of DockQ in pure Python. The updated version of DockQ is more portable, faster and introduces novel functionalities, such as automatic DockQ calculations for multiple interfaces and automatic chain mapping with multi-threading. These enhancements are designed to facilitate comparative analyses of protein complexes, particularly large multi-chain complexes. Furthermore, DockQ is now also able to score interfaces between proteins, nucleic acids, and small molecules.

**Availability and implementation:**

DockQ v2 is available online at: https://wallnerlab.org/DockQ.

## 1 Introduction

Biomolecular interactions between protein, nucleic acid, and ligands are crucial for all biological functions, including metabolism, gene expression, cell signaling, and immune response. Structural characterization of these interactions at the molecular level is important to understand basic biology, as well as disease mechanisms. Thus, elucidating biomolecular macromolecule structures has been the subject of immense research in previous decades. Experimental methods such as X-ray crystallography, NMR, and cryo-EM have determined the 3D structures of over 220 000 biomolecular structures (Berman *et al.* 2000). However, experimental determination of structures is difficult, time-consuming, and sometimes even impossible given experimental constraints. Also, 220 000 structures are a small number compared to the over 250 000 000 biomolecular macromolecules known to date (Suzek *et al.* 2015). Thus, there has been a massive drive to develop computational methods to close this gap by predicting the structural properties of biomolecular macromolecules.

To be able to compare the performance of computational methods it is important to have robust measures that can assess the quality of predicted models. Many such methods have been developed over the years, mostly for assessing monomer protein structures: MaxSub (Siew *et al.* 2000), GDT_TS (Zemla 2003), TM-score (Zhang and Skolnick 2004), and lDDT (Mariani *et al.* 2013). Lately, methods have been developed also for multimers: DockQ (Basu and Wallner 2016a), oligolDDT (Haas *et al.* 2019), or US-align (Zhang *et al.* 2022). In contrast to oligolDDT and US-align, which measure the overall structural similarity of multimers, DockQ assesses the quality of the interactions between chains in multimers. This is an advantage since the quality of these interactions might be obscured in the overall structural similarity.

Lately, next-generation prediction tools, such as RoseTTAFold-All-Atom (Krishna *et al.* 2024) and AlphaFold 3 (Abramson *et al.* 2024), have been released that can predict interactions between proteins, nucleic acids, and small molecules alike. In order to benchmark these new methods, it is important for a tool like DockQ to be able to evaluate interactions between all these types of molecules rather than forcing users to rely on multiple specialized tools.

We now introduce a new version of DockQ (v2). DockQ v2 is faster than the previous version, easier to install, and more portable. In this version, we include new functionalities: DockQ v2 can score multiple interfaces simultaneously in complexes with more than two chains and automatically detects the model-to-native chain mapping that maximizes the global quality of the complex. We also include the possibility of scoring interactions between proteins, nucleic acids, and small molecules. Even though other software can compare some of these molecules (Meli and Biggin 2020, Zhang *et al.* 2022, Collins *et al.* 2024), to the best of our knowledge, DockQ is the only tool that can seamlessly handle all these types of molecules.

In addition, we have also added interface clash detection, multithreading, transparent handling of mmCIF and gzipped files, the possibility to install it with *pip*, and the possibility to import it as Python module.

## 2 Method description

### 2.1 DockQ—assessing the quality of multimers

DockQ assesses the quality of a multimeric model by comparing it to a reference structure (typically an experimentally determined structure). The comparison is performed by assessing the similarity of the interfaces between protein chains. For a given protein-protein or protein-nucleic acid interface, the DockQ score is the average of the fraction of native contacts ($f_{\mathrm{nat}}$), scaled interface RMSD (iRMSD), and scaled ligand RMSD (LRMSD):

(1) $$\text{DockQ}=\frac{1}{3}\left(f_{\text{nat}}+\frac{1}{1+{\left(\frac{\text{iRMSD}}{a}\right)}^{2}}+\frac{1}{1+{\left(\frac{\text{LRMSD}}{b}\right)}^{2}}\right)$$

where a=1.5 and b=8.5 are scaling parameters for iRMSD and LRMSD, respectively, determined by maximizing the ability of DockQ to classify protein interfaces according to the CAPRI metrics (“Incorrect,” “Acceptable,” “Medium,” and “High quality”) (Collins *et al.* 2024). The advantage of the DockQ score is that it is a continuous score in the [0, 1] range, from incorrect to high quality, which facilitates method comparisons since distributions, instead of classifications, of model quality scores can be compared. This also makes it better suited for machine learning applications (Basu and Wallner 2016b).

To calculate the DockQ score using Equation (1), the $f_{\text{nat}}$, iRMSD, and LRMSD need to be calculated, below we describe how these measures are defined.

### 2.2 Fraction of correctly predicted reference contacts

The $f_{\text{nat}}$ measure is the fraction of correctly predicted reference or native contacts in the interface (Méndez *et al.* 2003). An interface is a set of interacting amino acids or nucleotides sitting in separate chains with at least one pair of heavy atoms within 5 Å of each other (4 Å in the case of peptides). A contact is correct if a pair of interfacial amino acids in the model are also interacting in the reference. The total number of correct contacts is then divided by the total number of contacts in the model interfaces. A problem with $f_{\text{nat}}$ is that it does not consider the number of false predictions. Thus, a model with a large number of false positives might still receive a fairly high $f_{\text{nat}}$. Also, models with clashes will obtain higher $f_{\text{nat}}$, taking it to the extreme, a model with all (x,y,z) coordinates at 0 will have $f_{\text{nat}}$=1.0. This is, in general, only a problem for models with many clashes. Therefore, the number of clashes is automatically detected and reported to the user in DockQ v2.

### 2.3 Interface RMSD

The interface RMSD, iRMSD, is the backbone RMSD of the interface residues. The backbone is defined as the N, CA, C, and O atoms for proteins and P, OP[1,2], O[2–5], and C[1–5] atoms for nucleic acids. Interface residues are defined as pairs of residues between protein and/or nucleic acid chains where any two heavy atoms are within 10 Å of each other (8 Å between pairs of Cβ atoms in case of peptides).

### 2.4 Ligand RMSD

The ligand RMSD, LRMSD, is the backbone RMSD of the ligand when superimposing on the receptor. The receptor is defined as the larger of the two chains by number of amino acids or nucleotides. If two chains are equal in size, the first will be considered the receptor. In the case of small molecule ligands, LRMSD is calculated on the position of all heavy atoms in the ligand when superimposing on the receptor interface (*pocket-aligned LRMSD*).

### 2.5 Workflow and new functionalities

DockQ used to be protein-only. We have now added the functionality to also score interactions involving nucleic acids, and small molecule ligands. In the case of small molecule ligands, only the pocket-aligned ligand RMSD (LRMSD) is reported, as commonly done in other methods (Bell and Zhang 2019, Meli and Biggin 2020). Since small molecules are not ordered like proteins and nucleic acids, we use NetworkX’s (Hagberg *et al.* 2008) graph matching functionality and report the solution with the lowest LRMSD when multiple matches are possible (e.g. symmetric molecules). Graph edges are calculated according to the covalent radii (Cordero *et al.* 2008) of the atoms.

DockQ reads the coordinates of a model and reference structure in PDB or mmCIF format, either uncompressed or compressed with *gzip*, to calculate the DockQ score [Equation (1)] for each interface in the reference structure. It returns the DockQ score for each interface as well as an overall *GlobalDockQ*, defined as the average of DockQ scores over all interfaces. Equivalent residues in the model and reference are mapped using sequence alignment (default) or by following the residue numbering.

For multimeric complexes with symmetric subunits, there is a need to find the optimal chain mapping, i.e. the chain mapping that maximizes the overall GlobalDockQ. This is also needed when multiple small molecules of the same type are being evaluated at once. The number of possible chain mappings grows with the factorial of the number of equivalent molecules, i.e. a homodimer (stoichiometry A2) has 2!=2 possible mappings, while a homooctamer (stoichiometry A8) has 8!=40320. The default behavior is that DockQ will identify equivalent subunits by sequence alignment (by molecule name in the case of small molecule ligands) and exhaustively permutate the chain mappings to find the one with the highest GlobalDockQ. It is also possible to provide a given chain mapping with the – –*mapping* option or to limit the search to a subset of native chains. The search is parallelized over multiple threads (default = 8) using LRU caching and with computationally intensive functions, such as distance calculations, implemented in Cython for speed.

DockQ v2 is released as a pip-installable Python package. This allows you to import and use DockQ as a module in other software.

## 3 Results

**Figure 1.**
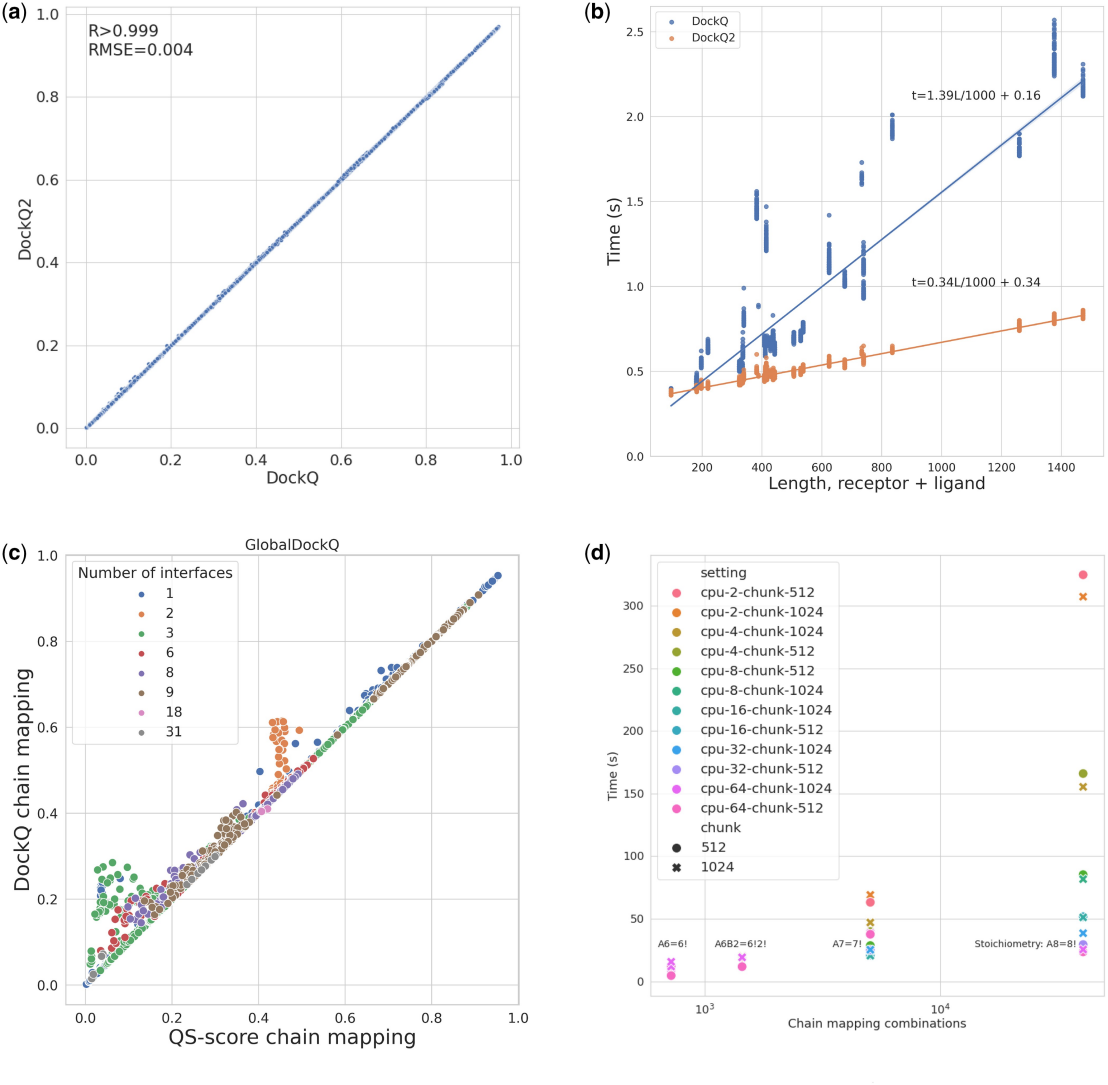
(a) We evaluated DockQ v2 against the old DockQ (v1.0) on a large benchmark of 17 049 models from 37 CASP15 targets. The DockQ scores are equivalent between the two versions. (b) The new version of DockQ is faster than the previous due to optimized implementation of computationally expensive functions in Cython, especially for larger complexes. (c) Comparison of methods to perform automatic mappings between model and native chains: the automatic mapping done in DockQ yields the same or better GlobalDockQ than when using mappings optimized by QS-score. (d) Time to exhaustively optimize the mapping between sets of chains for complexes with different stoichiometry using different number of cores (cpu) and chunk sizes (chunk), which is the number of work units sent to each cpu. The time complexity is linear with the number of chain mapping combinations, and can be proportionally cut down by increasing the number of parallel threads in DockQ.

To verify that DockQ v2 is able to reproduce the DockQ scores generated with the previous version, we compared DockQ scores calculated for 17 409 models from 37 CASP15 targets. The agreement is almost perfect, with an average error of 0.004 and a correlation of 1.000 (0.999996) between the new and the previous version (see Fig. 1a). Thus, the scores from both versions can be used interchangeably.

The updated version is generally faster, especially for larger targets, as shown in Fig. 1b. The linear portion of the time complexity is decreased approximately four times (4.08) compared to the previous version. This leads to significantly reduced runtimes in practice.

An important new feature is automatic chain mapping, which simplifies the problem of finding the optimal chain mapping between symmetric chains. Previously, the optimal chain mapping has either been ignored, assuming a one-to-one correspondence between the chains in model and reference, or had to be determined with some other software like the chain mapping routine in QS-score (Bertoni *et al.* 2017). The latter was used when calculating DockQ in the evaluation of multimer predictions in CASP15 (Studer *et al.* 2023). We compared the DockQ scores using the new automatic chain mapping to the DockQ scores obtained by using the mapping from the chain mapping routine in QS-score. In all cases, the automatic chain mapping in DockQ produces an identical or higher GlobalDockQ compared to using the mapping provided by QS-score (Fig. 1c). This is expected since the mapping using QS-score is optimized using QS-score, while the automatic chain mapping in DockQ is optimized on GlobalDockQ. To make the automatic chain mapping faster, it is parallelized on multiple threads, making it possible to exhaustively map a homomer with stoichiometry A8 (8!=40320 chain combinations) in around a minute on 8 threads or one with stoichiometry A7 (7!=5040 combinations) in <30 s (see Fig. 1d).

Taken together, we believe that this new, improved version of DockQ will become an essential tool for evaluating models of biomolecular complexes, regardless of the type of molecule they may include.
